# Juvenile Hormone Regulates Extreme Mandible Growth in Male Stag Beetles

**DOI:** 10.1371/journal.pone.0021139

**Published:** 2011-06-22

**Authors:** Hiroki Gotoh, Richard Cornette, Shigeyuki Koshikawa, Yasukazu Okada, Laura Corley Lavine, Douglas J. Emlen, Toru Miura

**Affiliations:** 1 Laboratory of Ecological Genetics, Graduate School of Environmental Science, Hokkaido University, Sapporo, Japan; 2 Anhydrobiosis Research Unit, National Institute of Agrobiological Sciences, Tsukuba, Japan; 3 Laboratory of Molecular Biology, University of Wisconsin-Madison, Madison, Wisconsin, United States of America; 4 Laboratory of Evolutionary Ecology, Graduate School of Environmental Science, Okayama University, Okayama, Japan; 5 Department of Entomology, Washington State University, Pullman, Washington, United States of America; 6 Division of Biological Sciences, University of Montana-Missoula, Missoula, Montana, United States of America; Michigan State University, United States of America

## Abstract

The morphological diversity of insects is one of the most striking phenomena in biology. Evolutionary modifications to the relative sizes of body parts, including the evolution of traits with exaggerated proportions, are responsible for a vast range of body forms. Remarkable examples of an insect trait with exaggerated proportions are the mandibular weapons of stag beetles. Male stag beetles possess extremely enlarged mandibles which they use in combat with rival males over females. As with other sexually selected traits, stag beetle mandibles vary widely in size among males, and this variable growth results from differential larval nutrition. However, the mechanisms responsible for coupling nutrition with growth of stag beetle mandibles (or indeed any insect structure) remain largely unknown. Here, we demonstrate that during the development of male stag beetles (*Cyclommatus metallifer*), juvenile hormone (JH) titers are correlated with the extreme growth of an exaggerated weapon of sexual selection. We then investigate the putative role of JH in the development of the nutritionally-dependent, phenotypically plastic mandibles, by increasing hemolymph titers of JH with application of the JH analog fenoxycarb during larval and prepupal developmental periods. Increased JH signaling during the early prepupal period increased the proportional size of body parts, and this was especially pronounced in male mandibles, enhancing the exaggerated size of this trait. The direction of this response is consistent with the measured JH titers during this same period. Combined, our results support a role for JH in the nutrition-dependent regulation of extreme mandible growth in this species. In addition, they illuminate mechanisms underlying the evolution of trait proportion, the most salient feature of the evolutionary diversification of the insects.

## Introduction

**Figure 1:**
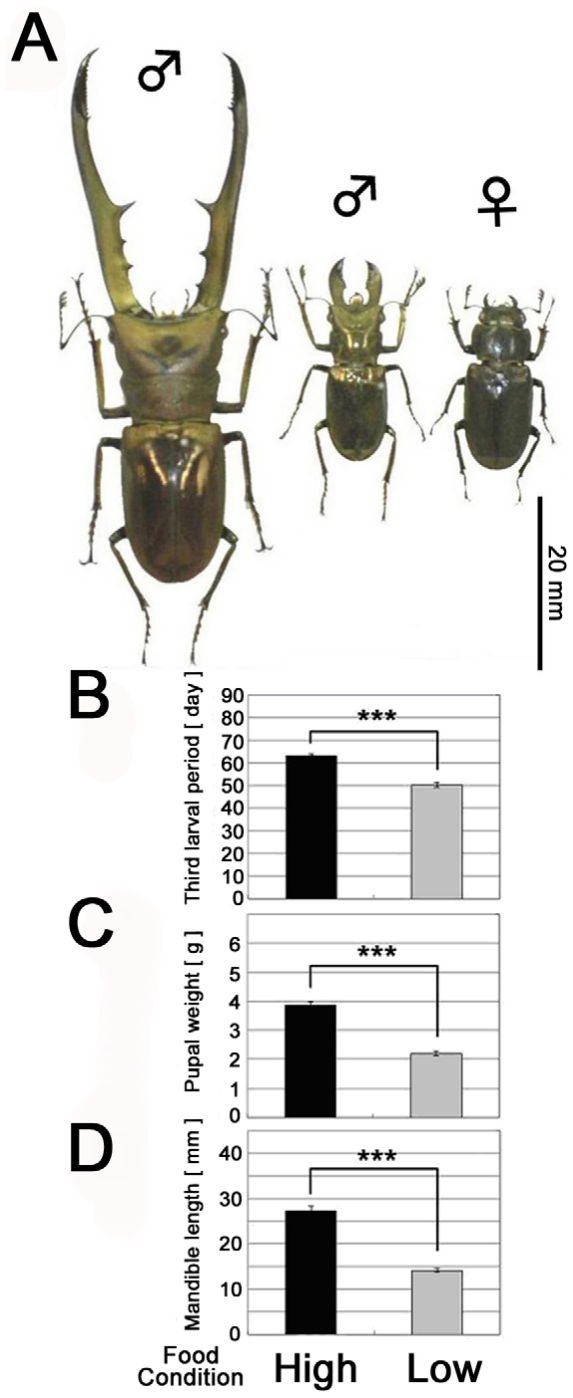
Intraspecific morphological variation in *Cyclommatus metallifer* and condition dependent effects of high and low food availability. (A) Intraspecific morphological variation in *Cyclommatus metallifer.* Large male (left), small male (center), and female (right) are shown. (B–D) Larval period duration, pupal weight and mandible length of stag beetle pupae reared under high and low nutritional regimes. (B) Males reared under high food conditions spent significantly more time as third instar larvae than males reared under low food conditions (P<0.001, student t-test). (C) Male stag beetles were significantly larger as pupae when reared under high food conditions (P<0.001, student t-test). (D) Adult mandible length was significantly longer in males reared under high food conditions (P<0.001, student t-test). The means ± SEs (N = 10) are shown.

Insects display an astounding variety of forms. Much of this diversity can be understood as the result of evolutionary changes in the proportional sizes of body parts [1], [2]. Stag beetles (Coleoptera, Lucanidae) exemplify the extremes that insect proportions can take. Male stag beetles wield some of the most extraordinary weapons of any animal, a pair of grotesquely enlarged mandibles, which they use in combat with rival males over females [3], [4]. Mandible size varies extensively among males (Fig. 1A), and this variation is driven by nutrition so that only the largest, best-fed individuals produce full-sized weapons (Fig. 1B–1D). Nutrition-sensitive growth is characteristic of all exaggerated animal structures yet studied (e.g. the ornaments and weapons of sexual selection [5]–[7]), and this has generated interest in the physiological mechanisms generating nutrition-dependent phenotypic plasticity and exaggerated, or disproportionate, trait growth [8], [9]. Yet, the mechanisms responsible for coupling nutrition with growth of these (or indeed any) insect structures remain largely unknown. In this study, we capitalize on vast among-individual variation in the extent of mandibular development, because males possess much longer mandibles in comparison with females, and because large males develop with longer mandibles than smaller males. These individual differences in the amount of mandible growth can be visualized as scaling relationships between mandible length and body size, and using both direct measurement of hormone titers in these divergent individuals, and augmentation of hormone levels, we demonstrate that during the prepupal period, juvenile hormone exerts quantitative effects on the relative amount of mandible growth. Our findings suggest that juvenile hormone may be an integral part of the mechanism generating nutrition-dependent (i.e. ‘conditional’) expression of exaggerated sexually selected structures in insects.

## Results and Discussion

### Histological background of mandible growth

**Figure 2:**
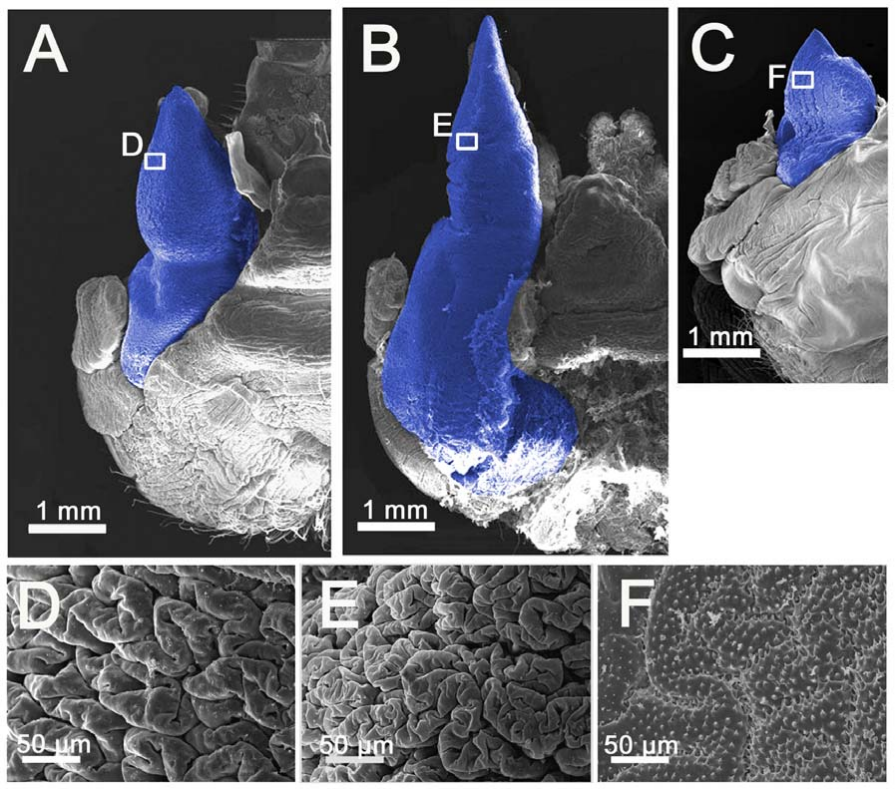
SEMs of the newly-formed stag beetle mandibles dissected from the prepupal cuticle immediately before pupation. (A–C) SEMs of the left side of newly-formed stag beetle heads and mandibles. (A) Small male, (B) large male, (C) female. Mandibles are indicated in blue. (D–F) Magnified images of the mandibular regions indicated by the white boxes in A, B, and C. The surface structures of the mandibles are different among the three types of individuals.

Stag beetles are complete metamorphic developers. Larvae undergo three instars and increase in weight over a combined feeding period of several months. At this point they have completed all growth in body size, and they begin the complex process of metamorphosis including gut purge, regression of larval-specific tissues, and rapid growth of adult structures. Eyes, wings, legs, genitalia and mouthparts all undergo rapid growth during this prepupa period, and in males, mandible growth is especially prolific at this time. Histological examination of the mandibles of both large and small males showed huge differences in epithelial proliferation during development (Fig. 2A and 2B). Under the larval cuticle of males, we found extensive folding of the epidermis and cuticle of the newly forming pupal mandibles. Among-male variation in the structure of these folds (density and depth of furrow, Fig. 2D and 2E) results in differences in mandible sizes of pupae. In contrast, for females with relatively small mandible sizes, we observed smooth mandibular epithelial surfaces lacking any folding in developing female prepupae (Fig. 2C and 2F). The complex folding resulting from epithelial growth and proliferation has also been reported in the development and formation of other exaggerated structures extending directly from the body walls such as the horns of horned beetles [10], [11] and the mandibles and nasus of termite soldiers [12], [13].

### Juvenile hormone regulates mandible growth

It is during the prepupa period that physiological signals are predicted to modulate the amount of growth in adult structures in response to nutrition. One proposed mechanism suggests that JH acts as a nutrition-sensitive regulator of trait growth [14], [15]. JH has long been known to influence alternative patterns of insect growth, including the seasonal onset of diapause and wing development in response to overcrowding [16]. JH is also thought to regulate the nutrition-dependent expression of traits such as caste-specific morphologies in ants and bees [17], and size-dependent production of horns in beetles [10]. Recently, Truman et al (2006) showed that JH is crucial for coupling nutrition with the growth of adult traits in the hornworm *Manduca sexta* [18].

**Figure 3:**
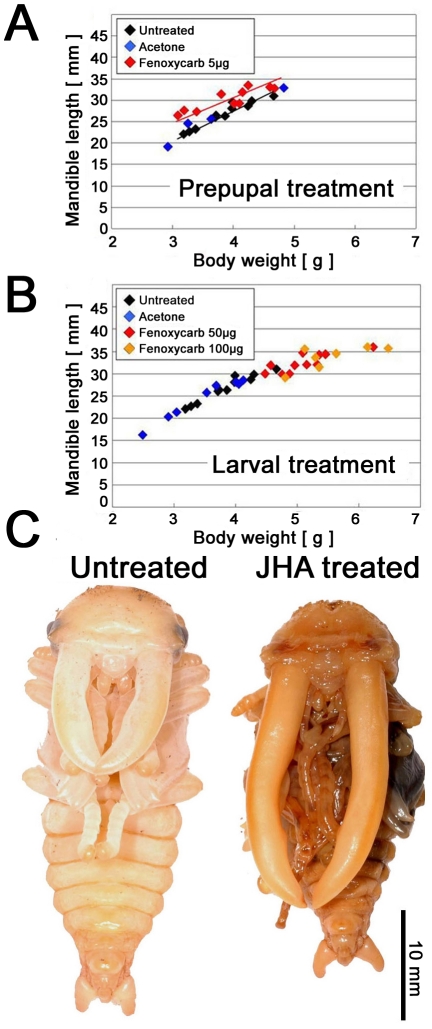
Effects of JHA treatment on male prepupae and larvae. (A, B) The relationship between body weight (*X*-axis) and mandible length (*Y*-axis) of male pupae treated with acetone or JHA at the prepupal period and the larval period. (C) Pupal phenotype of a JHA treatment at the early half of the prepupal period which has abnormally exaggerated mandibles (right), compared with untreated individual of the same weight (left).

In this study, we provide correlative evidence that JH regulates the exaggerated growth of male stag beetle mandibles. To test the role of JH during adult mandible development, we increased hemolymph titers of JH by application of the JH analog (JHA) fenoxycarb during both larval and prepupal development. Because growth in overall body size has ceased by the prepupal period, we predicted that perturbation of JH signaling during the prepupal period would affect growth of the adult structures without altering body size –i.e. it would alter the proportional sizes of body parts. As predicted, increasing JH signaling during the early prepupal period increased the proportional size of body parts, and this was especially pronounced in male mandibles (P<0.001, ANCOVA, Fig. 3A and 3C). In females, mandibular elongation was not observed regardless of the developmental stage, the type of JHA applied or the JHA application dose.

**Figure 4:**
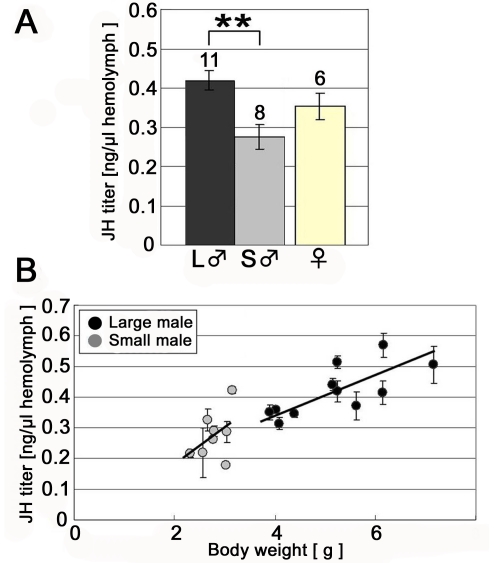
Comparison of JH titer between large and small males during the early prepupal period. (A) JH titer during the early prepupal period (mean ± SE). A significant difference in JH titer was found between large and small males. Numbers above each column denote the number of examined individuals. (B) The relationship between body weight and JH titer in males in the early prepupal period. The shaded circles represent the average value of the three measurements for a single individual with a standard error bar. Among individuals under high food conditions (i.e. large males), a significant positive correlation between weight and JH titer was found (P = 0.012, R^2^ = 0.55, Pearson correlation coefficient test). However, individuals reared under low food conditions (i.e. small males) showed no significant correlation between weight and hormone titer, possibly due to a much narrower range of body weights observed (P>0.1, R^2^ = 0.21, Pearson correlation test).

The direction of the response to ectopically expressed JHA in male beetles was consistent with the measured titers for JH during this same period (Fig. 4A and 4B). Significant differences in JHIII titers and body size were found between small and large males for the early prepupal period (small male: 0.282±0.033, large male: 0.413±0.034 ng/µl hemolymph, P<0.01, Tukey-Kramer; Fig. 4A and P = 0.012, Pearson correlation coefficient test; Fig. 4B). In contrast, perturbing JH during the end of the prepupal period did not influence exaggerated mandible growth (Fig. S1).

Regulation of condition-dependent trait proliferation by JH signaling depends on the precise deployment of JH [19]. Topical application of JHA to male stag beetle larvae prior to the prepupal period significantly prolonged the larval periods (Fig. S2) resulting in large pupae (Fig. 3B). However, these males did not show disproportionate growth of condition dependent traits as trait size and mandible size of these males scaled with that of normally reared males (Fig. 3B and S3). This result suggests that a high JH titer during the larval period contributes to increased total body size, while an increased JH titer at the early prepupal stage contributes to increased mandibular growth regardless of body size. We have shown that the mandibles of these male stag beetles are undergoing their maximal rate of growth during the early prepupal period (Fig. 2) and are expected to be most sensitive to perturbations in JH during this time. In addition, we found that male prepupal mandibles responded to perturbations of JH signaling more strongly than other adult male structures, suggesting that these are especially sensitive to this endocrine signal.

Taken together, these results suggest that in male stag beetles, JH signaling during the early prepupal period influences exaggerated mandible growth through the regulation of epidermal proliferation. To confirm this hypothesis, further analysis of the function of JH through JH knockdown experiments such as the artificial degradation of JH titers with anti-JH agents [20] or knock-down of JH signaling factors by RNAi [21], will be necessary.

Mandible size varies extensively among male stag beetles in response to nutrition. We have used a combination of experiments to manipulate JH and to directly measure JH titers during mandible development to establish hormonal differences. The results of this study suggest that a physiological mechanism generating nutrition-dependent phenotypic plasticity and exaggerated, or disproportionate, trait growth is JH signaling at a specific developmental window during mandibular growth. Our results are a first step in elucidating the physiological and genetic mechanisms responsible for coupling nutrition with growth of these exaggerated weapons of sexual selection.

## Materials and Methods

Additional Materials and Methods can be found in the Materials and Methods S1.

### Insect Husbandry

All individuals were reared in the laboratory under 24-hour darkness at 24±2°C. Stag beetles were purchased from Hercules-Hercules, Sapporo, Japan.

### Larval food manipulation

Third instar larvae were collected within 3 days of molting and were transferred into two experimental groups: a low nutrition group (reared in 120 ml cups) and a high nutrition group (reared in 430 ml cups). They were kept within an incubator under constant darkness at 25±0.5°C. Growth cup size affects the amount of available food and limits space for growth, affecting larval and adult body size. Growth between the two conditions was measured as time to pupation, pupal weight, and adult left-mandible length. Statistical analyses between the two groups were carried out with Student's t-test.

### Scanning electron microscopy (SEM)

The structure of the newly-formed mandibles of large males, small males, and females was observed by scanning electron microscopy (SEM) as described in Materials and Methods S1.

### Larval JHA treatment

The juvenile hormone analog (JHA) fenoxycarb (Wako Pure Chemical Industries Ltd., Japan), were diluted in acetone and topically applied to male third instar larva 3 days after molting in doses of 50 and 100 µg/larva). Ten µl of the JHA solution was applied to the dorsal thorax surface of each larva. Immediately after JHA application, the treated larvae were placed into individual 430 ml plastic cups filled with decaying wood flakes. They were reared in an incubator under 24-hour darkness at 25±0.5°C until pupation. Larval period (in days), weight (g) and mandible length of pupae and adults (prothorax width and left mandible length) were recorded. The control groups included an untreated group and a group that received an application of 10 µl of acetone.

### Prepupal JHA treatment

Male and female individuals in the early half of the prepupal period (staged as between pupal cell construction to termination of gut purge) and males in the late half of the prepupal period (staged from termination of gut purge to pupation) were treated with 10 µl of 5 µg fenoxycarb diluted in acetone as described above (Wako Pure Chemical Industries Ltd., Japan). Control groups were untreated, and acetone-only, early and late prepupae. Mandible length and total weight of all pupae was recorded. Statistical analyses for the relationship of mandible length to pupal weights among all groups were conducted using ANCOVA (JMP version 6.0, SAS Institute, Cary, NC).

### Hemolymph collection and JH extraction

Hemolymph was collected from the early prepupal periods of experimentally manipulated small and large individuals of both sexes. As mentioned, low food rearing containers with a limited food supply produce small beetles whereas larvae reared in large containers with over three times as much food develop into significantly larger individuals. Prepupae were anaesthetized on ice and hemolymph was collected by dissection at the base of the legs, with a glass Pasteur pipette. Aliquots of collected hemolymph were extracted by using methanol and iso-octane with 30 ng fenoxycarb (Wako Pure Chemical Industries Ltd., Osaka, Japan,) as an internal standard. See the Materials and Methods S1 for more detail.

### Liquid chromatography-mass spectrometry (LC-MS)

To quantify juvenile hormone titers in the stag beetle, the experimental methods of LC-MS were modified based on Westerlund et al. 2004 and Cornette et al. 2008 [22], [23]. 5 µl from each 20 µl extracted sample was separated on a 150×2 mm^2^ C18 reverse-phased column (YMC-Pack Pro C-18.5 µm, YMC Co., Ltd., Kyoto, Japan) protected by a guard column (YMC-Pack Pro, sphere ODS, YMC Co., Ltd., Kyoto, Japan) with gradient elution of water/methanol (0–15 min 80–100% methanol, 15–20 min 100% methanol) at a flow rate of 0.2 ml/min, utilizing an Agilent 1100 HPLC system with autosampler. Mass Spectrometry analysis was performed by electrospray ionization (ESI) in the positive mode on a microTOF-HS (Bruker Daltonics Inc., Billerica, MT) under the following conditions: the electrospray capillary was set at 4.5 kV and the dry temperature was 200°C. The nitrogen pressure was 1.2 Bar for the nebulizer and the drying gas nitrogen low-rate was 4 L/min. The Ionization of standard JH III generated [M+CH2O]^+^, [M+OH]^+^, [M+H]^+^ and [M+Na]^+^ ions. In hemolymph samples, [M+H]^+^ and [M+Na]^+^ were the major ions observed. To confirm these ions were JHIII generated, the HRMS (high resolution of mass spectrum) of these ions were compared with the HRMS of JHIII (C_16_H_26_O_3_) with DataAnalysis analysis software (Bruker Daltonics Inc., Billerica, MT). In order to diagnose the presence of additional JH homologs, JHI (m/z, 295, 317) and JHII (m/z, 281, 303) were also investigated. There were two candidate peaks for JH homologs in the chromatogram of the hemolymph extracts. One peak of m/z  = 295, 317, corresponded with JHI and the other m/z  = 267, 289 to JH III. HRMS analysis indicated that m/z  = 295, 317 was not JH I (C_18_H_30_O_3_) (error > 500 ppm), but m/z  = 267, 289 was closely matched with the exact-mass of JHIII (C_16_H_26_O_3_) (error <5 ppm). In addition, the retention time (RT) of the candidate m/z  = 267, 289 was the same as that of JHIII standard (Sigma, St Louis, MO). There were no clear peaks of m/z  = 281, 303 that correspond to JH II. Therefore, JH III was the only JH homolog found in *C. metallifer*. Quantification of JH III, fenoxycarb was therefore performed by monitoring the [M+H]^+^ and [M+Na]^+^ ions. A calibration curve of JH III was made, containing the same concentration of the internal standard fenoxycarb as for the hemolymph samples. The JH III titer from each sample was then calculated after analysis of the chromatogram data with QuantAnalysis software (Bruker Daltonics Inc., Billerica, MT).

## Supporting Information

Figure S1 **The relationship between body weight and mandible length of male pupae treated with acetone or fenoxycarb (JHA) at the late prepupal period.**
(DOC)

Figure S2 **Comparison of the duration of the third instar among males treated with fenoxycarb (JHA) or acetone only (mean ± SE).**
(DOC)

Figure S3 **The relationship between prothorax width and left-mandible length of adult males treated with acetone or fenoxycarb (JHA) during the larval period compared with normally reared males.**
(DOC)

Materials and Methods S1 **Materials and Methods for insect husbandry, scanning electron microscopy (SEM) and hemolymph collection and JH extraction.**
(DOC)
