# Supplementary material for: Juvenile Hormone Regulates Extreme Mandible Growth in Male Stag Beetles
Source: PLoS One. 2011 Jun 22;6(6):e21139. doi: 10.1371/journal.pone.0021139 (PMC3120829; doi:10.1371/journal.pone.0021139)
Supplement: Figure S1 — The relationship between body weight and mandible length of male pupae treated with acetone or fenoxycarb (JHA) at the late prepupal period. (DOC) [file pone.0021139.s001.doc]

**Supporting Information; Figure S1**

**
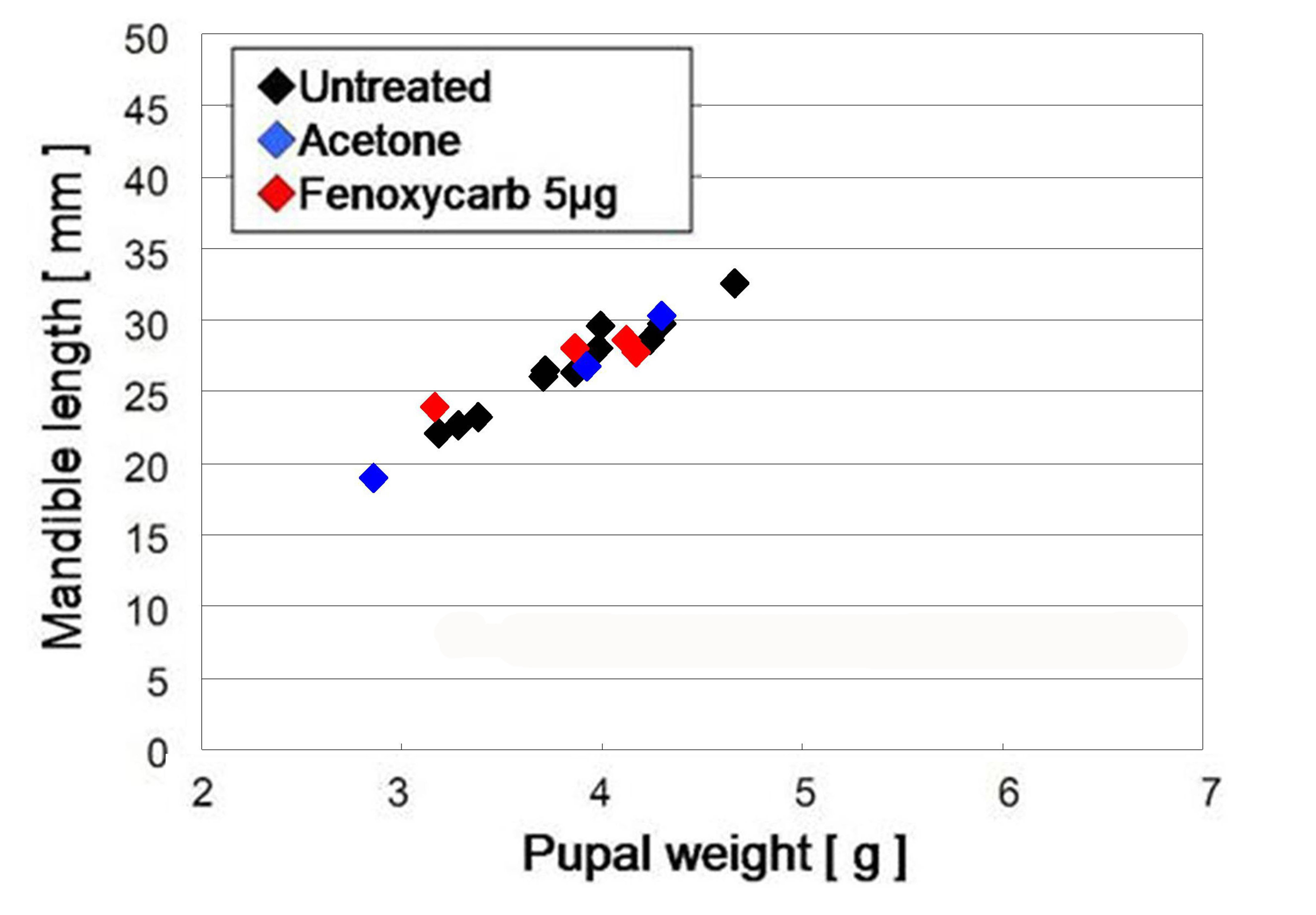
**

**Fig. S1.** The relationship between body weight and mandible length of male pupae treated with acetone or fenoxycarb (JHA) at the late prepupal period. Mandible elongation was not induced in either the acetone or JHA manipulation at the late prepupal period (P>0.1, ANCOVA).
