# Supplementary material for: Juvenile Hormone Regulates Extreme Mandible Growth in Male Stag Beetles
Source: PLoS One. 2011 Jun 22;6(6):e21139. doi: 10.1371/journal.pone.0021139 (PMC3120829; doi:10.1371/journal.pone.0021139)
Supplement: Figure S2 — Comparison of the duration of the third instar among males treated with fenoxycarb (JHA) or acetone only (mean ± SE). (DOC) [file pone.0021139.s002.doc]

**Supporting Information; Figure S2**


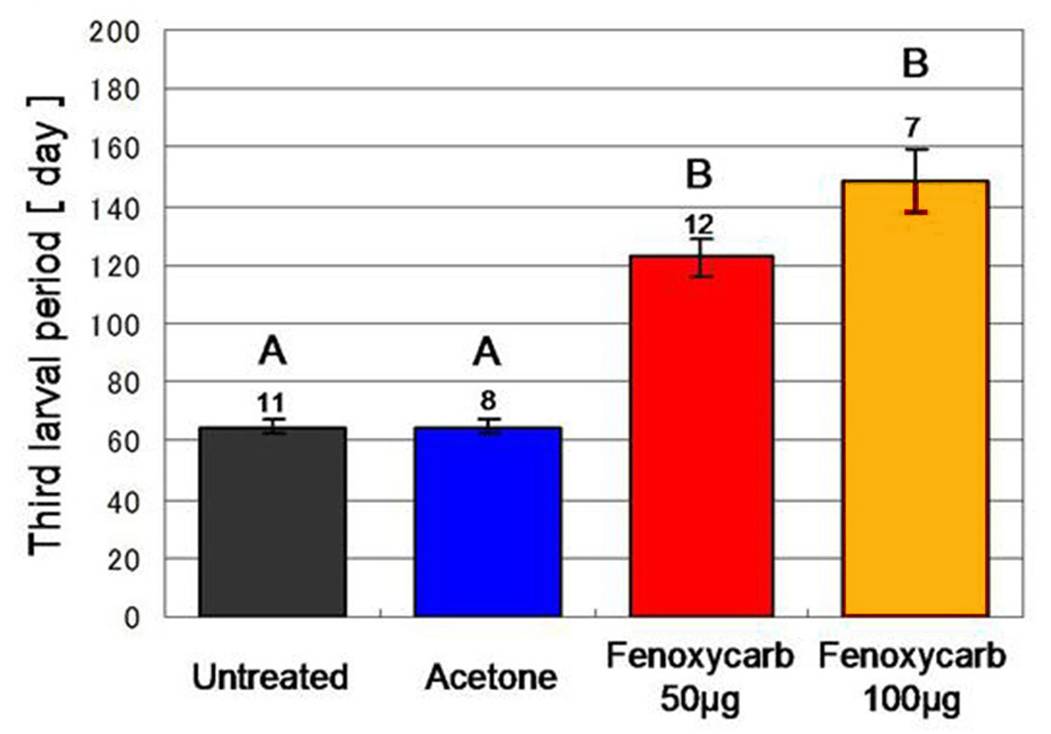


**Fig. S2.** Comparison of the duration of the third instar among males treated with fenoxycarb (JHA) or acetone only (mean ± SE). Application of JHA at the third larval instar significantly increased the larval period (P < 0.001, Tukey-Kramer). Letters above the bars denote significant differences among categories. Numbers on columns denote the number of individuals examined.
