# Supplementary material for: Juvenile Hormone Regulates Extreme Mandible Growth in Male Stag Beetles
Source: PLoS One. 2011 Jun 22;6(6):e21139. doi: 10.1371/journal.pone.0021139 (PMC3120829; doi:10.1371/journal.pone.0021139)
Supplement: Figure S3 — The relationship between prothorax width and left-mandible length of adult males treated with acetone or fenoxycarb (JHA) during the larval period compared with normally reared males. (DOC) [file pone.0021139.s003.doc]

**Supporting Information; Figure S3**


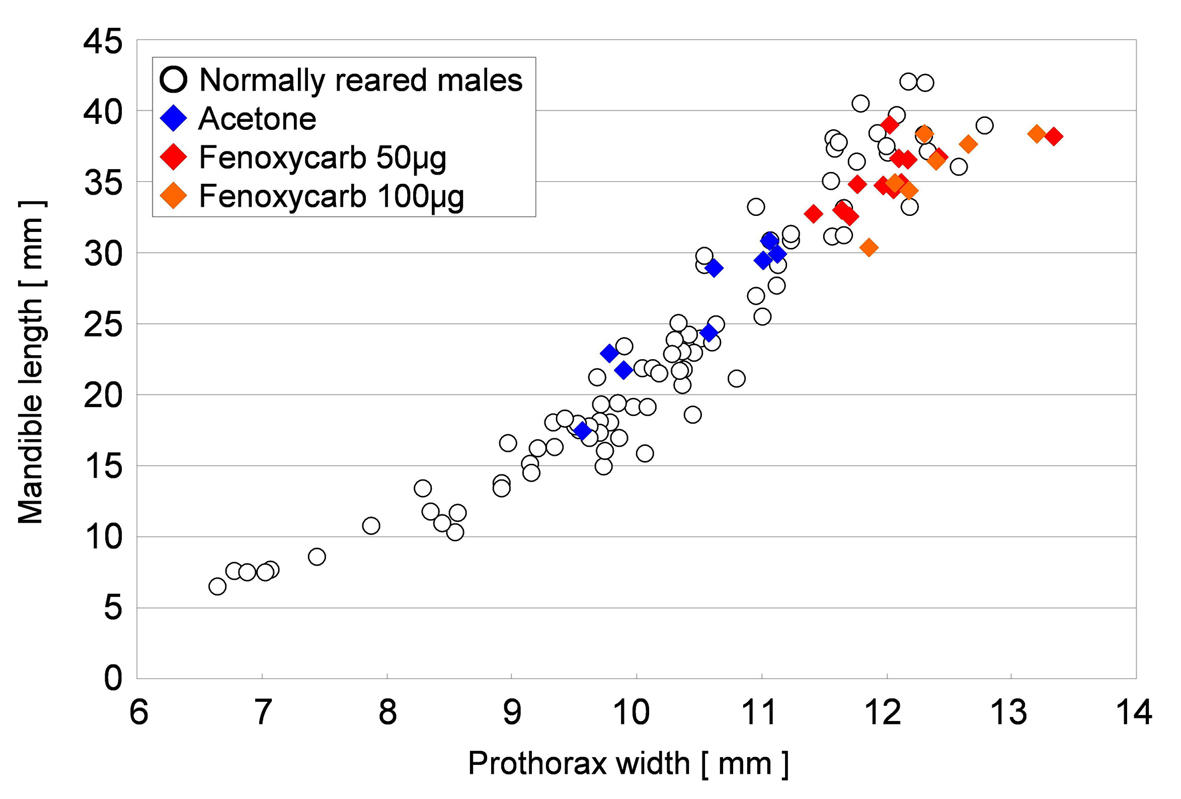


**Fig. S3.** The relationship between prothorax width and left-mandible length of adult males treated with acetone or fenoxycarb (JHA) during the larval period compared with normally reared males. Scatter plots of the JHA-treated males (red and orange rhomboids) are on the line of the scaling relationship of normally reared males (open circles). The JHA treatment on larvae did not change the normal scaling relationship of stag beetles.
