# Supplementary material for: Juvenile Hormone Regulates Extreme Mandible Growth in Male Stag Beetles
Source: PLoS One. 2011 Jun 22;6(6):e21139. doi: 10.1371/journal.pone.0021139 (PMC3120829; doi:10.1371/journal.pone.0021139)
Supplement: Materials and Methods S1 — Materials and Methods for insect husbandry, scanning electron microscopy (SEM) and hemolymph collection and JH extraction. (DOC) [file pone.0021139.s004.doc]

**Materials and Methods S1**

***Insect Husbandry***

All individuals of material species *Cyclommatus metallifer* were reared in the laboratory under 24-hour darkness at 24 ± 2 °C. Stag beetles were purchased from Hercules-Hercules, Sapporo, Japan. Breeding was performed in the laboratory as described below. One male and one to two females were placed together in a plastic container (Volume 850 ml), containing approximately 600 ml of decayed-wood flakes as a substrate (genus *Quercus;* Kuwagata-mat, Hercules-Hercules). Adults were fed with a commercial beetle jelly (Za-Okuwa Co.,Kawanishi, Japan). New jelly (16 g) was supplied once a week. Approximately, 30 breeding chambers were established. After two months, the adults were removed and eggs and larvae were gathered into a large plastic container filled with decaying wood flakes (container dimensions: 27 cm × 35 cm × 21.5 cm). The eggs and larvae were reared in a mass culture under 24 hour darkness at 24 ± 2 °C until the second instar when they were removed, individually maintained and observed for exact staging the third instar molt.

***Scanning electron microscopy (SEM)***

The structure of newly-formed mandibles in large males, small males, and females were observed by scanning electron microscopy (SEM). Prepupae that were within 3 hours of pupation were fixed in FAA fixative (formalin: acetic acid: ethanol = 6: 1: 16) for 2 hours. After fixation, the larval cuticle was removed from the heads with forceps under a binocular microscope (model SZ61, Olympus Global, Japan). The dissected tissues were then dehydrated through increasing concentrations of ethanol ending with a final rinse into *t*-butanol and then freeze-dried using a Freeze Dryer ES-2030 (Hitachi Global, Japan), and coated with gold ions with an Ion Sputter E-1010 (Hitachi Global, Japan). The detailed morphological characteristics of the mandibular structures were observed with a scanning electron microscope JSM-5510LV (JEOL Ltd., Tokyo, Japan).

***Hemolymph collection and JH extraction***

Hemolymph was collected from the early prepupal periods from experimentally manipulated small and large individuals of both sexes. As mentioned, low food rearing containers with a limited food supply produce small beetles whereas larvae reared in large containers with over three times as much food develop into significantly larger individuals. Prepupae were anaesthetized on ice and hemolymph was collected by dissection at the base of the legs, with a glass Pasteur pipette. Aliquots of collected hemolymph were stored in glass vials at -80 °C until use. The JH extraction protocol was modified from Cornette et al. 2008, as follows: 60 μl of hemolymph was blown into 540 μl methanol/iso-octane (1:1, v/v) containing 30 ng fenoxycarb (Wako Pure Chemical Industries Ltd., Osaka, Japan,) as an internal standard. The mixture was vortexed for 20 sec and allowed to stand at room temperature for 30 min, before centrifugation at 8500 g for 15 min. The iso-octane phase was transferred into a new glass vial, the methanol phase was vortexed and centrifuged at 10,000 g for 30 min and then combined with the iso-octane phase. The resulting mixture was stored at -20 °C or concentrated down to 20 μl and transferred to an auto-sampler vial for immediate analyses. This extraction process was performed in triplicate for each individual.
